# Left ventricular noncompaction in pediatric population: could cardiovascular magnetic resonance derived fractal analysis aid diagnosis?

**DOI:** 10.1186/s12968-021-00778-5

**Published:** 2021-07-08

**Authors:** Sylvia Krupickova, Suzan Hatipoglu, Giovanni DiSalvo, Inga Voges, Daniel Redfearn, Sandrine Foldvari, Christian Eichhorn, Sian Chivers, Filippo Puricelli, Grazia Delle-Donne, Courtney Barth, Dudley J. Pennell, Sanjay K. Prasad, Piers E. F. Daubeney

**Affiliations:** 1 Department of Paediatric Cardiology, Imperial College and Royal Brompton Hospital, Sydney Street, London, SW3 6NP UK; 2 Cardiovascular Magnetic Resonance Department, Royal Brompton Hospital, London, UK; 3 National Heart and Lung Institute, Imperial College, London, UK

**Keywords:** Left ventricular noncompaction, Fractal analysis, Children

## Abstract

**Background:**

Cardiovascular magnetic resonance (CMR) derived fractal analysis of the left ventricle (LV) has been shown in adults to be a useful quantitative measure of trabeculation with high reproducibility and accuracy for the diagnosis of LV non-compaction (LVNC). The aim of this study was to investigate the utility and feasibility of fractal analysis in children.

**Methods:**

Eighty-four subjects underwent CMR: (1) 28 patients with LVNC (as defined by the Petersen criteria with NC/C ratio $\ge$ 2.3); (2) 28 patients referred by clinicians for assessment of hyper-trabeculation and found not to qualify as LVNC (NC/C $\ge$ 1.8 and < 2.3); (3) 28 controls. The fractal scores for each group were presented as global and maximal fractal dimension as well as for 3 segments of the LV: basal, mid, and apical. Statistical comparison of the fractal scores between the 3 groups was performed.

**Results:**

Global fractal dimension (FD) was higher in the LVNC group than in the hyper-trabeculated group: 1.345 (SEM 0.053) vs 1.252 (SEM 0.034), p < 0.001 and higher in hyper-trabeculated group than in controls: 1.252 (SEM 0.034) vs 1.158 (SEM 0.038), p < 0.001. The highest maximum FD was in the apical portion of the LV in the LVNC group, (1.467; SEM 0.035) whereas it was in the mid ventricle in the hyper-trabeculated (1.327; SEM 0.025) and healthy groups (1.251; SEM 0.042). Fractal analysis showed lower intra- and interobserver variability than the Petersen and Jacquier methods.

**Conclusions:**

It is technically feasible to perform fractal analysis in children using CMR and that it is quick, accurate and reproducible. Fractal scoring accurately distinguishes between LVNC, hyper-trabeculation and healthy controls as defined by the Petersen criteria.

## Introduction

Left ventricular (LV) noncompaction (LVNC) is an important and widely recognized morphological trait consisting of prominent trabeculation, a thin compacted myocardial layer, and deep intertrabecular recesses in some cases [1–7]. The condition is highly heterogeneous with a range of physiologies including: a so-called benign form with normal LV function; dilated form with reduced LV function; hypertrophic form; hypertrophic and dilated form; restrictive form; and associated with congenital heart disease [8].

Diagnosis can be problematic due to the heterogeneity of LVNC and the naturally trabeculated nature of the LV. Diagnostic criteria for both echocardiography and cardiovascular magnetic resonance (CMR) are in current use [9–13] such as the echocardiographic techniques of Chin and Jenni and the CMR based techniques of Petersen. All essentially draw a demarcation line between normal and non-compacted cardiomyopathy cases and create a ratio that is used to denote abnormality. For example, this is defined as a maximum ratio > 2 in end-systole for the Jenni echo-based method, and $\ge$ 2.3 in diastole for the Petersen CMR method. But there is clearly a spectrum of trabeculation from normal through hyper-trabeculated (but normal) to non-compacted (NC; abnormal) [14] and it is clear that the demarcation line is to some degree attributed arbitrarily and these parameters are not able to discriminate LVNC especially when borderline values are obtained. There are alternatives such as Stollberger’s assessment of trabeculations [15] and Jacquier’s assessment of the mass [12] of NC myocardium as a percentage of the global mass, all of which have their own drawbacks. More recently, speckle-tracking-derived abnormal ventricular mechanics such as rigid body rotation have been proposed [13, 16, 17].

Fractal analysis is now commonly utilized in other branches of medicine [18, 19] and has been proposed as a novel methodology to assess LV trabeculation in the adult population with LVNC with high accuracy and reproducibility compared to the above methodologies [19–23]. Fractal dimension (FD) is able to describe the complexity of LV trabeculation by a single index in order to distinguish LVNC from healthy controls.

It is a semi-automated method, which allows assessment of how an object fills the space. It is used in nature to describe for example the complexity of landscapes [24] and it has been also explored in many areas of medicine for example identification and localisation of brain tumors, assessment of bone trabeculation helping to distinguish between osteoporosis and healthy bone, distinguishing different electroencephogram (EEG) patterns which correlate to brain activities and identification of early stages of breast cancer using mammography [18]. FD of computed tomography (CT) pulmonary angiograms correlates negatively with pulmonary vascular resistance index in patients with pulmonary hypertension and can be used to monitor the disease progression [19].

The aim of this CMR based study was to investigate the utility and feasibility of fractal analysis in the diagnosis of LVNC in the pediatric population.

## Methods

### Study population

This was a single-centre retrospective study of 84 children < 18 years undergoing a CMR scan between 2011 and 2018: 28 subjects with LVNC, 28 with hyper-trabeculation and 28 healthy controls.

All patients with suspected LVNC undergo a CMR scan as part of their initial clinical assessment in our dedicated pediatric cardiomyopathy service. For patients with inconclusive echocardiographic assessment regarding LV hyper-trabeculation and not meeting LVNC echocardiographic criteria, our departmental policy is to assess for possible LVNC using another modality which is preferentially CMR in our practice. The Petersen method is then used to distinguish patients into mutually exclusive groups as follows: patients are included in the LVNC group if the largest noncompacted/compacted (NC/C) layer ratio is measured $\ge$ 2.3 in diastole in any segment in any of the 2-, 3- or 4-chamber views and in the hyper-trabeculation group if the ratio is $\ge$ 1.8 and < 2.3 [9].

For the purposes of this study, individuals were included in the control (healthy) group, if the CMR scan revealed completely normal findings. The indication for these scans was non-diagnostic echocardiogram, uncertainty about the anatomical structures on echocardiography, syncope or chest pain with low pre-scan probability of being cardiac in origin.

The study was approved by the Local Research Ethics Committee. Parents or guardians signed a written consent.

### Cardiovascular magnetic resonance

Contiguous standard short axis cines were performed on a dedicated 1.5 T CMR scanner (Sonata/Avanto, Siemens Healthineers, Erlangen, Germany), with full myocardial coverage and retrospective electrocardiographic (ECG) triggering from breath-held, balanced, steady-state free-precession cines. Typical cine frame time was 40 ms, TE 1.1.ms, field-of-view 320–360 mm frequency encoding (FE) × 240-280 mm phase encoding (PE) directions, acquired spatial resolution 1.7–1.9 mm in FE × 1.7–1.9 mm PE direction with 8 mm slice thickness and 2 mm gap, additionally parallel imaging with 1.7 acceleration factor was used. All images were analyzed with validated software (cvi42, Circle Cardiovascular Imaging Inc., Calgary, Alberta, Canada). Volumes and mass measurements were indexed to body surface area (BSA) and the following parameters were calculated: indexed LV end-diastolic volume (LVEDVI; mL/m^2^), indexed LV end-systolic volume (LVESVI; mL/m^2^), LV ejection fraction (LVEF). Papillary muscles and trabeculations were included in the global myocardial mass (and excluded from blood pool) in-line with our published departmental protocol [25]. For the purposes of trabeculation analysis, LV trabeculation was measured using 3 methods: Petersen, Jacquier and fractal analysis. The Petersen method was used to identify the 3 studied groups. Essentially, all 3 long-axis cines were assessed. The largest non-compacted to compacted layer ratio was calculated in diastole where the observer thought the ratio would be highest. This could either be mid-ventricular or apical depending on the LV anatomy of particular child. Several measurements were made for each case and highest obtained value was recorded as non-compacted/compacted myocardial ratio.

Patients were included in the normal, hyper-trabeculated and non-compacted groups if the ratio was < 1.8, $\ge$ 1.8 and < 2.3, and $\ge$ 2.3, respectively. The Jacquier method was based on measurement of the percentage of noncompacted mass from total LV mass and value above 20% was considered as diagnostic for LVNC. Non-compacted mass was calculated as the subtraction of compacted mass from total LV mass. Compacted mass was acquired by outlining the endocardial border while including the papillary muscles and excluding the trabeculations. If the trabeculations could not be defined, they were included into trabeculation measurement.

**Fig. 1:**
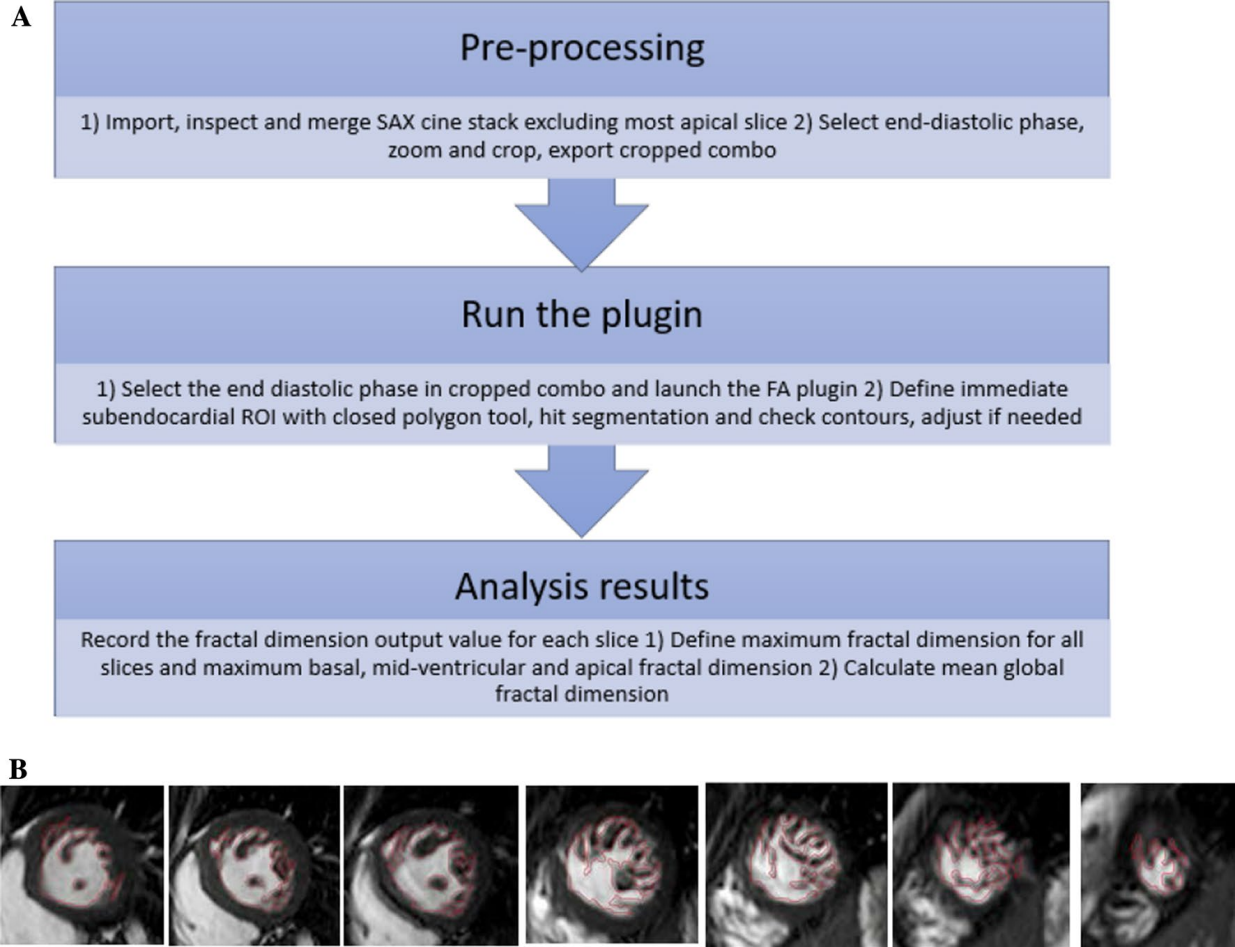
**A** Cardiovascular Magnetic Resonance (CMR) Trabecular Fractal Analysis using OsiriX Plugin (MATLAB graphical user interface). **B** Example of segmentation using fractal plug-in in a patient with left ventricular non-compaction (LVNC)

Fractal analysis was performed on the end-diastolic frames of each short-axis slice in the LV stack using a dedicated semi-automated MATLAB graphical user interface (MathWorks Inc, Natick, Massachusetts, USA) [20] with full details as previously reported [26]. The analysis was carried out in steps also detailed in Fig. 1A. The first step was pre-processing: thumbnails of the short axis stack were merged into a *combo* followed by cropping of the *combo* at end-diastole and exporting to the main dashboard, subsequently fractal analysis was performed: Region of interest was applied carefully along the subendocardium in each slice, followed by automatic segmentation, manual checking and fine-tuning [26]. In cases where the delineation of the trabeculations was not found to be accurate, the advanced segmentation parameter characterizing contour was adjusted to a contour 5 mm rather than 10 mm. The outcome of this step can be seen in Fig. 1B. Finally, automatic calculation of the FD using standard box-counting method separately in each slice was performed. The most apical slice was automatically excluded from the calculation by the software as it is prone to partial volume artefact.

For the purposes of the study, the following parameters were identified:

1. Average fractal dimension of all short axis slices was recorded as **global FD**
2. **Maximum FD** was described as highest FD value obtained from a single slice within the short axis cine stack
3. Maximum FD value from a single slice was also recorded separately within the basal, mid-ventricular and apical slices

Fractal analysis as above took approximately 3–5 min per patient.

Intra- and interobserver variability was assessed by assessing the LV trabeculations using fractal analysis in 30 individuals (10 with LVNC, 10 with hyper-trabeculation, 10 normals) using all 3 methods by the same observer (SK for intraobserver variability, performed with one-month temporal separation between repeat analyses) and by different observer (SH for interobserver variability) blinded to the first measurements.

### Statistical analysis

For statistical analysis, a p-value < 0.05 was considered significant. Statistical analysis was performed in R for Windows version 3.5.2 (https://cran.r-project.org/bin/windows/base/). Descriptive data were expressed as mean (standard error of mean, SEM) except where otherwise stated. Patient group characteristics were non-normally distributed and compared by the Wilcoxon signed-rank non-parametric test. Correlation between continuous variables was assessed with Pearson’s correlation coefficient. Fractal dimension was compared between the three study populations using Student’s t-test. For the fractal method, area under the receiver operating characteristics (ROC) curve (AUC) was calculated using the method described by Robin et al. [27]. Optimal threshold values for diagnosing LVNC were calculated as the Youden Index J, where J = max [sensitivity c + (specificity c − 1)], with c representing ranges over all possible criterion values. Intra and interobserver agreement for paired measurements from fractal analysis were evaluated using the Bland–Altman method [28]. Intraclass correlation coefficient (ICC) was used to compare reproducibility between the various methods. Variability of binary outcome data for each method using established cut-off values was assessed using Fleiss’ Kappa test for concordance. For all methods, sensitivity, specificity, positive and negative predictive values with exact 95% CI was reported.

## Results

### Patient characteristics

There were no significant differences between the 3 groups (LVNC, hyper-trabeculation and healthy controls) for age, weight, BSA and gender (Table 1). The table also shows the Petersen NC/C ratios. Mildly decreased LVEF was observed in 5 patients with LVNC, 1 patient has significantly depressed LVEF. Eleven out of 28 patients in the LVNC group underwent genetic testing and mutations were detected in 6. These were PKP2, TTNT2, MYH7, RYR2, NKX2-5 mutations and 8p23.1 microdeletion syndrome. In the hypertrabeculation group, 2 children had genetic testing and no mutation was detected. In the LVNC group 3/23 children had definite late gadolinium enhancement (LGE) in the myocardium (20 had no significant LGE and LGE CMR was not performed in remaining five patients) and none of the patients in the hypertrabeculation group had myocardial fibrosis (LGE was assessed in 17 of 28 patients). None of the patients in the normal group had gadolinium assessment.

**Table 1:** Demographics data of the 3 studied groups (LVNC, hypertrabeculation and normal controls), N = 84

Variable	LVNC (N = 28)	Hypertrabeculation (N = 28)	Normal (N = 28)
Age (years)	14(4)	12(3) p = 0.098*	13(3) p = 0.512**
Male/Female	16/12	18/10 p = 0.594*	18/10 p = 1.000**
Weight (kg)	51(18)	47(16) p = 0.238*	49(17) p = 0.422**
Height (cm)	156 (22)	154(16) p = 0.550*	156(19) p = 0.617**
BSA (m^2^)	1.48 (0.35)	1.41(0.31) p = 0.321*	1.45(0.35). p = 0.461**
Fractal global	1.345(0.053)	1.252(0.034) p < 0.001*	1.158(0.038) p < 0.001**
Fractal max	1.475(0.041)	1.346(0.027) p < 0.001*	1.259(0.043) p < 0.001**
Petersen	2.8(0.4)	1.8(0.2) p < 0.001*	1.1(0.1) p < 0.001**
Jacquier	0.35(0.10)	0.19(0.07) p < 0.001*	0.14(0.08). p = 0.012**
LVEDVI (ml/m^2^)	67(15)	67(11) p = 0.658*	65(11) p = 0.870**
LVESVI (ml/m^2^)	27(11)	24(7) p = 0.566*	22(5) p = 0.522**
LVSVI (ml/m^2^)	40(9)	43(8) p = 0.209*	43(8) p = 0.837**
LVEF (%)	61(11)	65(7) p = 0.189*	66(5) p = 0.594**
LV mass (g/m^2^)^a^	96(18)	82(15) p = 0.002*	69(10) p < 0.001**

Mean (SEM)

*Wilcoxon test comparing LVNC and hypertrabeculation groups

**Wilcoxon test comparing hypertrabeculation group and normal controls

^a^Trabeculations were included in the global LV mass for our volumetric analysis, therefore the difference in LV mass index between groups is significant

*BSA* body surface area, *LV* left ventricular, *LVEDVI* left ventricular end-diastolic volume index, *LVEF* left ventricular ejection fraction, *LVESVI* left ventricular end-systolic volume index, *LVNC* left ventricular non-compaction, *LVSVI* left ventricular stroke volume index

### Fractal dimension

**Fig. 2:**
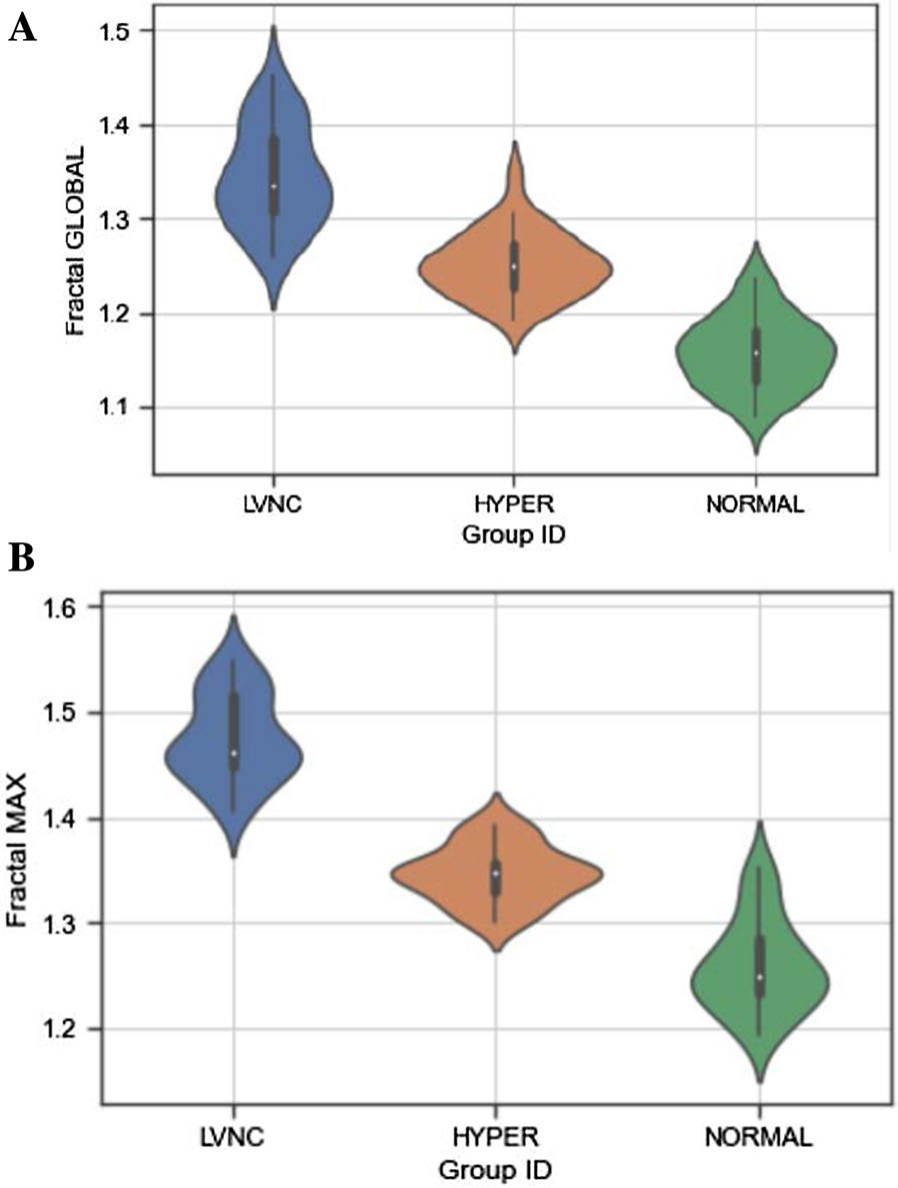
Global (**A**) and maximum fractal dimension (FD) (**B**) in all studied subjects. Violin plots show the distribution of global (**A**) and maximum FD (**B**) in the LVNC, hyper-trabeculated group and controls. There was no overlap between the LVNC and hyper-trabeculated/ normal groups for mean maximum FD. Both global and maximum FD were higher in the LVNC group than in the hyper-trabeculation group (p < 0.001) and controls (p < 0.001). Mean ± SEM

Global FD shown in Fig. 2 was highest in the LVNC group: 1.345(0.053) when compared to the hyper-trabeculation group: 1.252(0.034), p < 0.001 and healthy controls: 1.158 (0.038), p < 0.001. Maximum FD was higher in the LVNC group: 1.475 (0.041) than in the hyper-trabeculation group: 1.346 (0.027), p < 0.001 and controls: 1.259 (0.043), p < 0.001. There was no overlap in the mean maximum FD between the LVNC and hyper-trabeculated/ normal groups but there was an overlap between hyper-trabeculated and normal groups (Fig. 2). All LVNC subjects had a maximum FD > 1.4 and in all hyper-trabeculation subjects < 1.4.

**Fig. 3:**
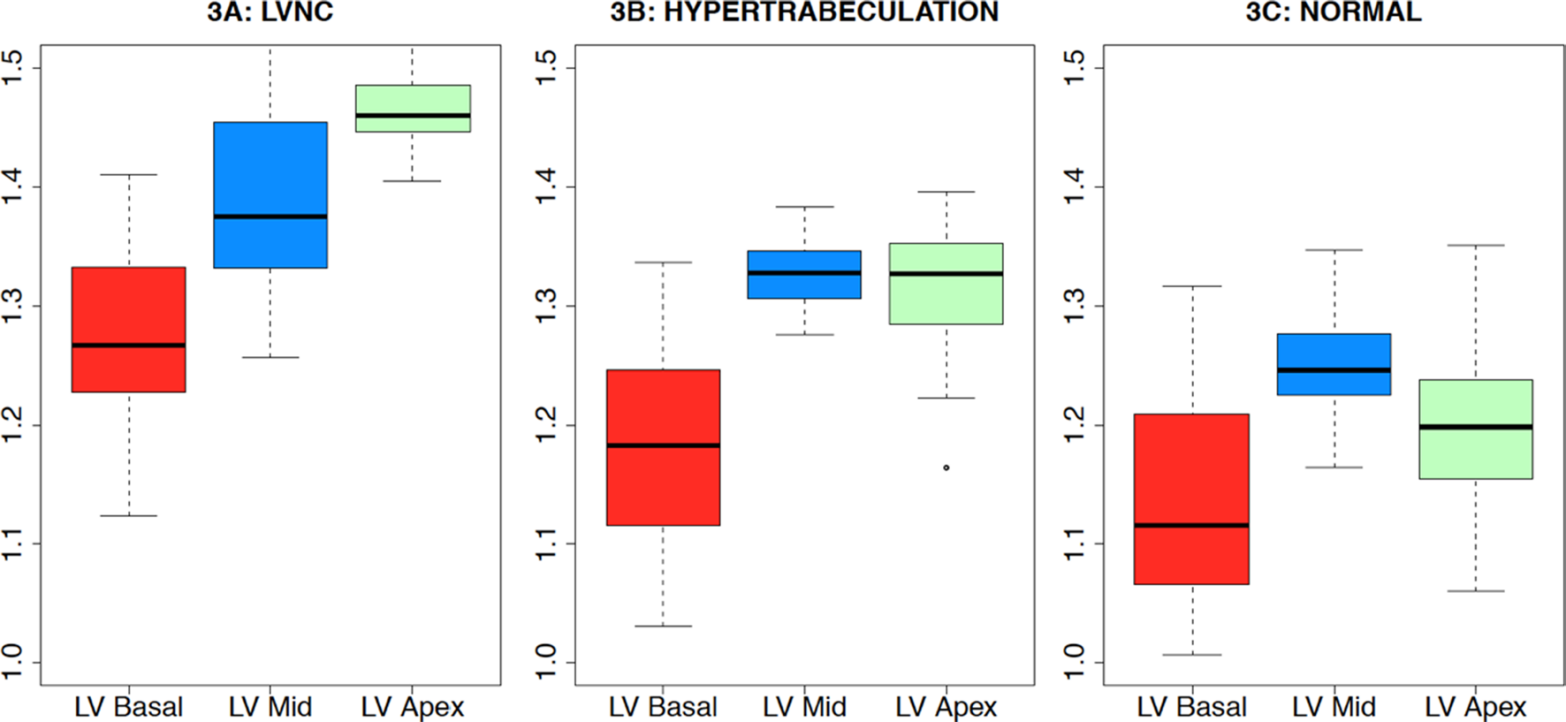
Distribution of maximum fractal dimension (FD) within the LV for the LVNC, hyper-trabeculation and normal groups. The LVNC group (**A**) had a different pattern of maximum FD distribution when compared to the hyper-trabeculated group (**B**) and normal (**C**). There was an increase in maximum FD from base to apex in the LVNC group [maximum FD at apex 1.467 (0.035)] whereas the hyper-trabeculated and normal groups showed highest maximum FD in the middle third of the LV [1.327 (0.025), 1.251 (0.042), respectively]

When maximum FD was compared between all 3 groups at the level of the LV basal, middle and apical thirds separately (Fig. 3), the values were significantly higher in all thirds in the LVNC group when compared to the hyper-trabeculated group and significantly higher in the hyper-trabeculated group when compared to the normal group in the middle and apical thirds only (Table 2).

**Table 2:** Maximum fractal dimension in the LV basal, mid and apical third in all 3 study groups

	LVNC	Hypertrabeculation	Controls
Basal third	1.274 (0.078)	1.179 (0.082) p < 0.001*	1.140 (0.088) p = 0.078**
Mid third	1.394 (0.082)	1.327 (0.025) p = 0.001*	1.251 (0.042) p < 0.001**
Apical third	**1.467 (0.035)**	1.316 (0.058) p < 0.001*	1.195 (0.074) p < 0.001**

Highest maximum fractal dimension in each study group is highlighted in bold

Mean (SEM)

*Wilcoxon test comparing LVNC and hypertrabeculation groups

**Wilcoxon test comparing hypertrabeculation and control groups

There were different patterns for the maximum FD distribution from base to apex in the LVNC group on the one hand and in the hyper-trabeculated and healthy control groups on the other. In the LVNC group, the maximum FD was in the LV apical third and lowest in the basal third. By contrast, in the hyper-trabeculated and healthy control groups the reverse was true with the highest maximum FD being in the middle part of the LV (highlighted in bold in Table 2).

### Reproducibility

**Fig. 4:**
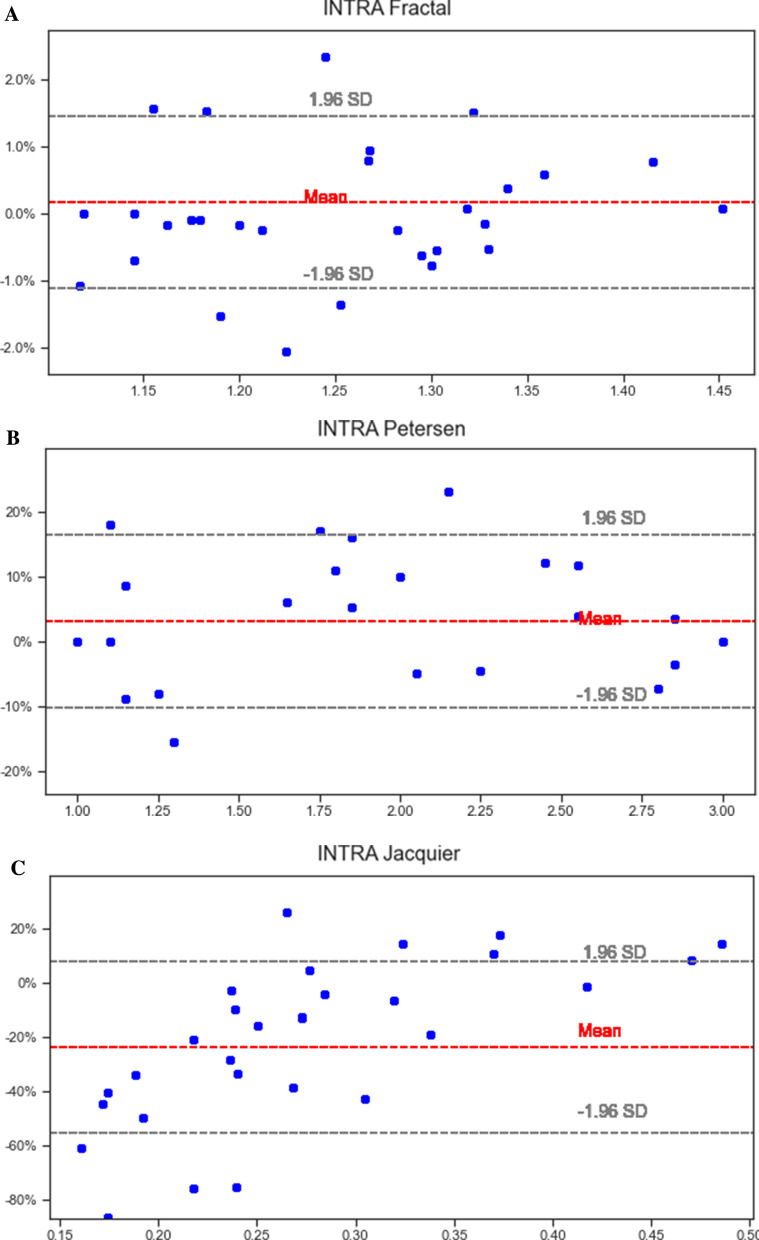
Bland Altman graphs showing the intraobserver variability for all 3 methods—Fractal analysis (**A**), Petersen (**B**) and Jacquier (**C**). Fractal analysis showed the best intraobserver variability from all 3 methods [intraclass correlation coefficient (ICC) 0.99, 95% CI 0.98, 1.00]. The Petersen method had more intraobserver variability than the fractal method (ICC 0.95, 95% CI 0.90, 0.98) and the Jacquier method showed the worst intraobserver variability (ICC 0.82, 95% CI 0.63, 0.92)

Fractal analysis showed the best intraobserver variability from all 3 methods (intraclass correlation coefficient (ICC) 0.99, 95% CI 0.98, 1.00). The Petersen method had more intraobserver variability than the fractal method (ICC 0.95, 95% CI 0.90, 0.98) and the Jacquier method showed the worst intraobserver variability (ICC 0.82, 95% CI 0.63, 0.92). Bland Altman plots are shown in Fig. 4. Fractal analysis also showed the lowest bias (0.00) with lowest lower and upper bounds (− 0.03, 0.03, respectively) when compared to other methods.

**Fig. 5:**
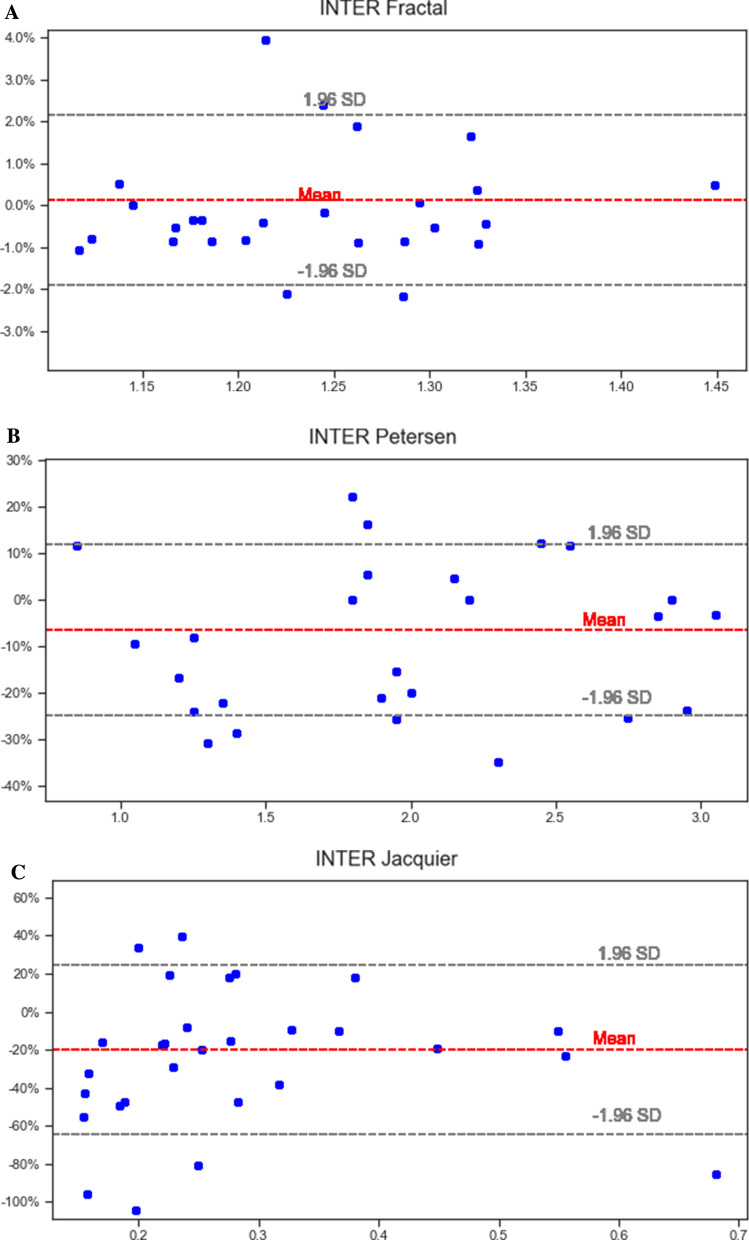
Bland Altman graphs showing the interobserver variability for all 3 methods—Fractal analysis (**A**), Petersen (**B**) and Jacquier (**C**). Interobserver variability was better using the fractal method (ICC 0.97, 95% CI 0.95, 0.99), and considerably more using the Petersen (ICC 0.92, 95% CI 0.83, 0.96) and Jacquier methods (ICC 0.74, 95% CI 0.45, 0.88)

Interobserver variability was lower using the fractal method (ICC 0.97, 95% CI 0.95, 0.99), and considerably higher using the Petersen (ICC 0.92, 95% CI 0.83, 0.96) and Jacquier methods (ICC 0.74, 95% CI 0.45, 0.88). Fractal analysis showed very low bias (0.002) and limits of agreement (− 0.050, 0.054) (Fig. 5).

## Discussion

This is the first study of the utility of fractal analysis in LVNC in a pediatric population. It shows for the first time in this age group that: (1) FD was significantly higher in the Petersen-determined LVNC group than in a hyper-trabeculated group, (2) FD was significantly higher in the hyper-trabeculated group than in healthy controls, and (3) fractal analysis was more reproducible than the Petersen and Jacquier methods.

One of the most advantageous features of the FD is its inherent capability to describe the complexity of different objects by single index. In LVNC, FD helps to compute how much of the two-dimensional image space is filled by the contour. It quantifies complexity of geometric patterns. The resulting FD is a unitless quantitative index of how completely the object fills space. FD increases with increased structural complexity. LV FD uses a box-counting method on CMR short-axis cine stacks. As the range of possible FD for a fractal set is dictated by its dimension, the endocardial borders visualized in two-dimensions, are more complicated than simple straight lines, so their FD must be > 1, but because they do not fill two-dimensional space completely, their FD must be < 2. Therefore, the range of possible FD for an endocardial border is between 1 and 2. In LVNC, trabeculations create irregular endocardial border, therefore should generate a higher FD when compared to normal hearts.

In contrary to Petersen method, FD is independent of compacted wall thickness which is possibly a confounding factor causing overdiagnosis or underdiagnosis in hypertrophied hearts when there is hypertrabeculation but is more accurate in LVNC with wall thinning (small value in denominator) [22]. Therefore, as FD assess purely the extent of trabeculations, it is not expected to change if the hearts dilate or become dysfunctional. Furthermore, we observed in our study, that the maximum and global FD are quite heterogenous in the borderline group of patients with NC/C ratio between 2.2 and 2.4 confirming the fundamental differences between Petersen and FD measurements favouring FD analysis in diagnosis of LVNC.

Recently fractal analysis has been used in adults to assess the complexity of LV trabeculation in a 2-dimensional setting in order to distinguish LVNC from healthy black and white controls [19]. Fractal analysis also has utility in other cardiac diseases for example in asymptomatic carriers of hypertrophic cardiomyopathy. It has been shown, that FD is a pre-clinical marker in adult sarcomere gene mutation carriers of hypertrophic cardiomyopathy as these individuals express increased trabecular complexity in terms of global FD and maximum apical FD when compared to genetically negative healthy controls [21]. In a large cohort of patients with atherosclerosis, reference values for FD in the adult population have now been established [22]. Being the first report of FD analysis in children and including a normal subjects’ group, our work may also serve as a reference of FD values in a pediatric cohort. A recent study reported that maximal apical FD combined with myocardial strain are promising markers for differentiation between LVNC from DCM [29].

The great advantage of fractal analysis, especially in this population, is that it does not require any special sequences and thus does not prolong the scanning time. The whole analysis is performed off-line using the short-axis cine stack, which is always part of the clinical CMR scan and analysis is less user-dependent compared to the Petersen or Jacquier methods.

Several groups studied genetics of the LVNC and it is now clear that no specific gene ontology exists. LVNC has been reported in patients with dilated cardiomyopathy, hypertrophic cardiomyopathy, restrictive cardiomyopathy, arrhythmogenic right ventricular cardiomyopathy, channelopathies, bicuspid aortic valve, aortopathy, congenital heart defects and in the pediatric population it was also associated with rare genetic syndromes, mitochondrial cardiomyopathy, and Barth syndrome [30, 31]. In our study, substantial number of patients in the LVNC group (11 out of 28) underwent genetic testing and six different mutations were detected including PKP2, TTNT2, MYH7, RYR2, NKX2-5 mutations and 8p23.1 microdeletion syndrome. Two children from the hypertrabeculation group had genetic testing and no pathogenic mutation was detected. Nevertheless, it is not possible to discharge patients from cardiomyopathy service based on negative genetic testing. Recent retrospective genetic study in 327 patients reported that although a detected mutation was associated with major adverse cardiac events, 68% of LVNC patients were free of a pathogenic mutation. Although not being the primary finding of the study, genetic data of our cohort confirms the heterogenous nature of LVNC and possible overlap with other cardiomyopathies [32]. Another feature of CMR is the assessment of myocardial fibrosis, and LGE could offer prognostic information. In our cohort 3 out of 23 children in the LVNC group had definite LGE enhancement and no significant fibrosis was detected in subjects with hypertrabeculation.

### Diagnostic accuracy

As the accuracy of fractal analysis depends on the spatial resolution and image quality of the underlying cine images, it was important to ascertain if the high reproducibility achieved in the adult population (intraobserver ICC 0.98, interobserver ICC 0.97 [20]) could be replicated in children. We were able to demonstrate that the diagnostic power was as high as in adult population (intraobserver ICC 0.99, interobserver ICC 0.97) despite including small children with clearly smaller hearts than the adults (20 children were less than 10 years old). However, it is necessary to note that all cines included into this study were SSFP cines and no real time sequences were analyzed. Occasionally, when children are not cooperative in our institution, we use either real time sequences or reduced signal averages, and it would require further research to analyze if these allow accurate measurement of FD.

The Petersen method was found to have worse reproducibility than fractal analysis and this might be explained by the fact that this technique although very fast and not requiring additional sequences, is very much reader dependent. Low reproducibility could also be contributing to the fact that Petersen criteria is usually not able to discriminate pathology from physiologic adaptation when the NC/C is borderline [22]. The Jacquier method is very time consuming and showed poor reproducibility in our study. Moreover, using this technique, there was a large overlap even between LVNC patients and the healthy group. It appears that the reason for this is user-dependent delineation of the trabeculation in each slice. Just for comparison the trabeculation is calculated automatically by the FD software and although checking by the operator is important, only rarely are minor adjustments required.

Our results show that the maximum FD should be considered as an alternative and perhaps preferable parameter for establishing the diagnosis of LVNC in children as there was no overlap between the LVNC group and the hyper-trabeculated or normal group. Never less, for patients maximum FD of 1.4 which divides both groups, we propose to use a combined approach of using clinical parameters such as family history, ECG, LV volumes, LVEF, genetics data, Petersen criteria and LGE. Global FD calculated as the mean of FDs in every slice of the LV is less able to group the patients correctly which may have been caused by the fact that the basal slices have less trabeculation even in the noncompacted group. A similar distribution pattern was also found in the adult population [20]. Whereas FD increases from the basal LV towards apex with the greatest FD in the apical third of the LV, this does not apply for controls and interestingly not even in hyper-trabeculated individuals. The latter 2 groups had the greatest FD at the mid-ventricular LV level due to the papillary muscles causing larger FD inherently involved into the FD calculation. Never less, global FD if significantly increased (over 1.35) could serve as a strong discriminative parameter for the LVNC when compared to hypertrabeculated group.

In the future it will be important to further ascertain the clinical utility of fractal scoring by in particular evaluating if fractal scoring is strongly associated with outcome and biomarkers. In this context, recent work by Wang et al. reported that increased apical fractal dimension (≥ 1.325) was an independent predictor of all-cause mortality and aborted sudden cardiac death in patients with hypertrophic cardiomyopathy after adjustment for the European Society of Cardiology predictors and LGE [33].

## Limitations

In this study we utilized the Petersen method as the current “gold standard” for CMR diagnosis of LVNC. We acknowledge that the many drawbacks to this method make it imperfect—in fact this was the very rationale for this study. Ultimately, we do believe an alternate automated method with low intraobserver and interobserver variation such as fractal scoring would be preferable. However, in presenting for the first time an alternative we felt the current standard should be utilised to ascertain the groupings.

## Conclusion

We have shown that fractal analysis is a promising and reproducible imaging method for the diagnosis of LVNC in the pediatric population. Maximum FD in particular may serve as a good marker for distinguishing between children with LVNC and normals and those with hyper-trabeculation (but not noncompaction). This may serve as a useful adjunct in the diagnosis of LVNC in children, either as a sole method or complementary to the Petersen method. The clinical utility now needs to be assessed in a long-term outcome study acknowledging that there may be a benign group of LVNC. Further research in this area is warranted.

## Data Availability

The datasets used and analysed during the current study are available from the corresponding author on reasonable request.

## Abbreviations

BSA: Body surface area
CMR: Cardiovascular magnetic resonance
CT: Computed tomography
ECG: Electrocardiogram
EEG: Electroencephalogram
FD: Fractal dimension
LGE: Late gadolinium enhancement
LV: Left ventricle/left ventricular
LVEDVI: Left ventricular end-diastolic volume index
LVEF: Left ventricular ejection fraction
LVESVI: Left ventricular end-systolic volume index
LVNC: Left ventricular non-compaction
LVSVI: Left ventricular stroke volume index
NC: Non-compacted
NC/C: Non-compacted/compacted ratio
